# The relationship between semen parameters in processed and unprocessed semen with intrauterine insemination success rates

**DOI:** 10.4274/jtgga.galenos.2018.2018.0089

**Published:** 2019-02-26

**Authors:** Leila Mollaahmadi, Afsaneh Keramat, Ashraf Ghiasi, Mozhgan Hashemzadeh

**Affiliations:** 1 Student Research Committee, School of Nursing and Midwifery, Shahroud University of Medical Sciences, Shahroud, Iran; 2 Center for Health Related Social and Behavioral Sciences Research, Shahroud University of Medical Sciences, Shahroud, Iran

**Keywords:** Intrauterine insemination, sperm parameters, pregnancy rate

## Abstract

**Objective:**

To evaluate the relationship between semen parameters and intrauterine insemination (IUI) success rates.

**Material and Methods:**

This retrospective study was conducted during a 4-year period (2011-2015) on the medical records of 350 couples admitted to the infertility center of Beast Hospital in Tehran. The participants’ data such as age, duration of infertility, semen parameters [including volume, concentration, motility, normal morphology and total motile sperm count (TMSC)] before and after sperm processing, as well as the IUI results were extracted from the patients’ records. Only the first IUI cycle of the couples was considered. The main outcome criterion for the IUI success was serum positive beta human chorionic gonadtotropin 14 days after IUI. The collected data were analyzed using the Mann-Whitney U test, chi-square, and Fisher’s exact tests.

**Results:**

The overall pregnancy rate for each couple was reported as 23.42% (82/350). There was no significant difference in the mean age of the couple and infertility duration between the groups who achieved pregnancy and those who failed. The two groups showed no significant differences in pre and post processing of semen parameters (including volume, concentration and TMSC). Sperm motility and normal sperm morphology before and after sperm processing were significantly different between the two groups, respectively (p=0.023 before sperm processing and p=0.032 after) (p=0.032 before sperm processing and p=0.007 after).

**Conclusion:**

Sperm motility and normal sperm morphology have an effect in IUI success.

## Introduction

Infertility is defined as the failure to conceive after one year of regular intercourse, without the use of contraceptives. Ten to fifteen percent of couples are infertile (1). The use of techniques such as intrauterine insemination (IUI) in conjunction with controlled ovarian stimulation have increased the hope for pregnancy in infertile couples. IUI is the first-line treatment, a non-invasive and cost effective procedure for the treatment of infertile couples and is performed by inserting a higher concentration of prepared sperms into the uterine cavity (2,3).

Semen analysis is the first step in the evaluation of male infertility because male factors account for 25 to 40% of infertility cases (4). In these assessments, semen characteristics including volume, sperm concentration, sperm motility, and normal sperm morphology are usually evaluated. The standard value of semen analysis that is accepted by the World Health Organization (WHO) 2010 is;

 1. Volume: 1.5 mL, 

2. pH: 7.2, 

3. Sperm concentration: 20 million/mL or more, 

4. Total motility (progressive and non-progressive): 40%, 

5. Normal sperm morphology: 4% (5,6).

In the literature, several semen parameters have been described in association with IUI results. There are controversial findings about the best evaluation method of semen analysis as a predictor of IUI success (7,8,9). Ruiter-Ligeti et al. (10) showed that semen processing significantly improved most of the sperm parameters. In a study by Basirat et al. (11), the presence of sperm progressive motility before semen processing was found to be the most important factor in predicting IUI outcomes (11). In contrast, Luco et al. (12) reported none of the pre or post processing semen analysis parameters considered to be predictors of pregnancy in couples undergoing IUI (12). 

Numerous studies reviewed the effect of total motile sperm count (TMSC) on IUI success rates. There is still discrepancy on a reliable TMSC cut-off to predict IUI outcomes (13,14,15). Some reserchers found a significant decrease in pregnancy rates when the total motile sperm count was less than 10 million (14,16,17). On the contrary, others stated that the TMSC did not appear to be a predictor of IUI success (15). Xiao et al. (18) showed that a low TMSC on the day of IUI did not reduce the chance of pregnancy in couples that underwent IUI (18).

There are conflicting results and there is no consensus on semen parameters associated with IUI success (7,10,12,13). In the present study, our objective was to investigate the relationship between semen parameters and IUI success in couples referred to Beast Hospital Infertility Center.

## Material and Methods

This retrospective study was performed over a 4-period from 2011 to 2015 using the medical records of 350 couples admitted at the infertility center of Beast Hospital in Tehran.

Couples who underwent IUI during the 4-year study period at this center and had one year or more primary infertility were enrolled in the study. Only the first IUI cycle of the couples was included in the study. The female partner had regular menstrual cycles and normal pelvic ultrasonogrophy and at least one normal and open uterine tube in hysterosalpingography or laparoscopy.

Regardless of the cause of male infertility, all male partners who were candidates for IUI were included. They had normal semen analysis or one or two of the sperm parameters were below the values established by the WHO (2010). Both pre- and post-processing semen results were available.

Incomplete data regarding the pregnancy outcome or missing data on pre and post processing sperm parameters results were excluded. All pregnancies were confirmed with serum positive beta human chorionic gonadtotropin (β-hCG) 14 days after IUI. Demographic data such as the age of couple, duration of infertility, semen parameters before and after sperm processing, and also the IUI results were extracted from the patients’ records. 

The subjects under study were divided into two groups, those who achieved pregnancy and those who failed, and the two groups were compared.

### Sperm preparation

Semen samples were collected from the male partners following 3-5 days of sexual abstinence by masturbation in sterile plastic containers at the infertility clinic. Liquefaction was performed at room temperature for 30 minutes. The initial analysis of semen parameters (volume, sperm count, sperm motility, sperm with normal morphology) was performed manually according to WHO guidelines (2010). The TMSC was calculated using the folowing formula:

Count (million/mL) × motility (% as a decimal fraction) × volume (mL)

Semen samples were prepared using the standard swim-up techniques. Each specimen was covered with double volume Ham’s F10 medium (Merck, Germany) and warmed at 37 °C (99 °F) for 45 min. The top layer, which now contained the most active sperm, was suspended in the medium (centrifugation was performed at 2500 g for 5 min). After discarding the supernatant, the residual substance was washed with the medium (centrifuging for 5 min at 2500 g) and then the supernatant was discharged. The isolated fraction of motile sperm was diluted in 0.5-1 mL of the same preparation medium and incubated until the time of insemination. Ater the processing procedures, the sperm analysis was reevaluated and the TMSC was recalculated.

### Insemination method

For all women, 50-100 mg clomiphene citrate was administered from the third day of menstruation for 5 days and human menopausal gonadotropins (75 to 150 units) was injected intramuscularly on days 6, 8, and 10. When at least one 18 mm follicle was detected under ultrasonography, hCG (5000-10000 IU) was injected intramuscularly. Thirty six hours later, 0.5-1 mL of the processed sperms, which was prepared only from fresh semen, was injected using a Wallace catheter very slowly for 3 minutes into the fundus of the uterus. The catheter was withdrawn very slowly and the patient then rested in the supine position for 30-45 minutes. Luteal phase support was provided with a 400 mg daily progesterone suppository, and 14 days after IUl, the serum β-hCG was measured to confirm pregnancy.

### Statistical analysis

The SPSS software (version 22) was used to record all data. The results are presented as mean ± standard deviation (SD). Comparison between variables (female and male age, duration of infertility, semen parameters) was performed using the Mann-Whitney test because data distribution according to the Kolmogorov-Smirnov test was not normal. Categorical variables were evaluated using the chi-square and Fisher’s exact test. Statistical significance was accepted as p<0.05.

## Results

A total of 350 IUI cycles were analyzed. The overall pregnancy rate per couple was 23.4% (82/350). A comparison of the demographic data between the groups who did and did not achieve pregnancy is shown in Table 1.

The mean (±SD) female age in the pregnant and non-pregnant groups was 28.68±4.14 and 29.25±5.20 (range, 19-48) years, respectively. The mean (±SD) male age was 33.01±5.41 years in the pregnant group and 32.59±4.78 years in the non-pregnant group (range, 21-49 years). As can be noted, there were no significant differences in the female or male ages among both groups (Table 1).

Table 2 shows the outcome of IUI (pregnancy rate) for the different female age groups. 

Regarding the age of the female, patients were divided into four age groups as follows: <25 years, 25-29 years, 30-34 years, and >35 years.

Out of 82 pregnancies that occurred, 37 (45.1%) were achieved in the age group of 25-29 years, and 7 (8.5%) were achieved for the age of 35 years and over. However, according to the chi-square test, no significant correlation was found between female age and IUI success (p=0.578).

In addition, the two groups did not differ statistically for the duration of infertility (Table 1).

Table 3 shows the comparison between pre and post processing semen analysis parameters between the pregnant and non pregnant groups.

There was no significant difference in semen parameters including sperm volume, sperm concentration and TMSC before and after sperm processing between the two groups (Table 3).

TMSC was divided into four groups: <1×10^6^, 1-4.99×10^6^, 5-10×10^6^, and >10×10^6^.

Pregnancy rates for the subgroups of pre and post processing TMSC are compared in Table 4.

The highest pregnancy rate occurred in TMSC of over 10 million and the lowest pregnancy occurred in TMSC of under 1 million. However, there was no significant relationship between pregnancy rate and TMSC pre and post processing semen analysis (p=0.503 and p=0.761, respectively).

Only sperm motility and normal sperm morphology before and after sperm processing were significantly associated with pregnancy rates between the two groups (p=0.023 before sperm processing and p=0.032 after) (p=0.032 before sperm processing and p=0.007 after, respectively) (Table 3).

## Discussion

In the literature, pregnancy rate after IUI has been reported differently and it was dependent on several female and male factors (7,8,13,14). The results of our study showed that the pregnancy rate with IUI was 23.4% for each couple (82/350). This is similar to the results of (23.5%) Sinha et al. (19) and is in line with other studies (15,20,21). 

In some studies, female age was shown to be an important predictor factor of IUI success (8,21,22). Yousefi and Azargon (21) showed that with an increase of patients’ age, the pregnancy rate decreased, thus in their study, most pregnancies with IUI were observed in patients aged under 35 years (21). In the study of Ghaffari et al. (23), a negative relationship between female age and IUI outcome was shown.

The age-related decline in female fertility is attributable to the reduction of ovarian reserve and the aging of the reproductive system (20,24,25). In our study, there was no significant difference regarding the mean age of the women in the pregnant and non pregnant groups (p=0.508) (Table 1). We also compared pregnancy rates for different female age groups. Out of 82 pregnancies, 37 (45.1%) occurred for those aged between 25-29 years and only 7 (8.5%) occurred in women aged over 35 years (Table 2). However, the results of the present study are similar to the results of Basirat et al. (11), Koyun Ok et al. (14), Ganguly et al. (26), and Yildirim et al. (27) who failed to show a significant correlation between female age and IUI success (p=0.578). We believe that this is more likely due to the small population in our study. Sharma et al. (28) published a study showing that with an increase in male age, the fertility rate was reduced. In this study, there was no significant difference in the age of males between the pregnant and non-pregnant groups. This finding is in agreement with several studies (11,19,27,29). 

The reports of some studies indicated that with the increase in the duration of infertility, the chance of pregnancy decreased, which is probability attributed to the increased age of patients (23,27,30). Yavuz et al. (31) found that the pregnancy rate in couples with a period of infertility of less than 6 years was 2.33 times higher than those with infertility problems for over 6 years. The results of our study are similar to those of other studies that found no significant association between the duration of infertility and IUI success (7,8,14,20,26).

Although several studies reported the effect of semen parameters on IUI success (7,10,14,32), Luco et al. (12) failed to indicate such a relationship. A lack of agreement exists about the best semen parameters that can predict the possibility of pregnancy after IUI (13,32,33). Ruiter-Ligeti et al. (10) evaluated the impact of semen processing on sperm parameters and pregnancy rates after IUI. They found that semen processing led to significant increases in most sperm parameters such as the percentage of motile sperm and forward sperm progression (10).

Zhao et al. (34) published a retrospective study showing that pre and post processing sperm motility were independent factors that affected pregnancy rates. Our results agree with several studies that showed that sperm motility significantly influenced pregnancy rates after IUI (p=0.023 before sperm processing and 0.032 after) (7,8,10,21,31).

The importance of sperm morphology alone to predict IUI results before or after sperm preparation is controversial. Some researchers found that sperm morphology in male infertility was not a prognostic factor in IUI success (12,35). In contrast, Aboutorabi et al. (36) showed that in comparison with the other semen parameters, normal sperm morphology before and after semen processing had higher sensitivity and specificity and was more effective in predicting IUI outcomes (36). Lemmens et al. (37) concluded that none of the sperm parameters had a direct association with IUI success, but sperm morphology ≤4% could contribute to IUI success. Our result demonstrated that normal sperm morphology was significantly associated with pregnancy rates (p=0.032 before sperm processing and 0.007 after). These results also confirm the findings achieved by Jellad et al. (8) and Kdous et al. (32)

Wiser et al. (13) published a study to design a model to predict IUI success. This model included all basic sperm characteristics: sperm concentration (million/mL) × volume (mL) × motility (%) × morphology (%) and showed that the total motile normal sperm count was a more reliable criterion to predict IUI success. 

The minimum of TMSC recommended by authors varies in different studies, and is reported to be between 0.8 to 10×10^6^ (8,14,16,17,32). Tan et al. (38) discussed the predictive value of postwashed TMSC on IUI success. They showed that TMSC was an independent predictor, and to achieve statistically pregnancy rate after IUI, at least 0.5×10^6^ or greater TMSC was needed (38). A “linear by linear” relationship between post-processsing TMSC and IUI sucess was observed in Koyun Ok et al. (14). Tournays (39) declared that the TMSC could predict pregnancy failure more than pregnancy success; when the TMSC is lower than 1 million, in vitro fertilization should be suggested (39). In contrast, Hassan et al. (15) evaluated the impact of both pre and post processing TMSC on pregnancy rates and showed that pregnancy rates following IUI were unaffected by TMSC. In the present study, we found no significant difference for TMSC between the groups who did and did not achieve pregnancy. 

Pregnancy rates for subgroups of pre and post processing TMSC were compared and in this regard, most pregnancies observed were with a TMSC of more than 10 million and the lowest pregnancy rate was observed in TMSC under one million. It should be noted that we had very few subjects with TMSC under 10 million. However, it is difficult to determine the effects of TMSC on IUI outcomes at these levels of subjects. Our results are consistent with those of several studies (15,18,23) that found no significant relationship between TMSC and IUI success (pre p=0.503 and post p=0.761 semen processing). Similar to our findings, Zadehmodarres et al. (22), Koyun Ok et al. (14), and Kdous et al. (32) demonstrated that sperm concentration before and after preparation had no significant effect on IUI success (14,22,32). However, Dadkhah et al. (30) did find such a relationship. 

There are few articles about the correlation of the volume of inseminated sperm with IUI success (14,23). Study by Ghaffari et al. (23) showed the influence of semen volume in predicting IUI success. In 2013, Koyun Ok et al. (14) evaluated the effect of low semen volume on pregnancy rates. In agreement with our study, no significant relationship was shown between semen volume and IUI outcomes.

Variations and inconsistencies in the literature concerning predictive factors for IUI sucess can be attributed to the heterogeneity of the studied populations, the small size of the study population, the method of using statistical tests, and the lack of prospective clinical studies. Besides, differences in the correct use of standard criteria for sperm preparation, injection techniques, ovulation induction regimens, reporting method, inadequate care for women after sperm injection, and lack of adequate education should not be neglected (21,37,40,41). 

The principal limitation of this study is that it was a retrospective study. Data were collected from information previously registrated in the patients’ records. No documents were available about the number of follicles and serum hormones such as follicle-stimulating hormone, luteinizing hormone, and anti-mullerian hormone. However, in this study we only included male factors in order to make the differences in sperm parameters more significant. In comparison with other studies, the population evaluated in our study was small and may not be adequate to achieve statistical significance for some parameters such as age and TMSC. Hence, larger prospective, randomized, controlled clinical studies are recommended. Another limitation is that clinical pregnancy was only defined as serum-positive β-hCG two weeks after IUI and sonography results were not recorded for the observation of a gestational sac and fetal heart rate in the patients’ records. Considering the fact that the goal of infertility treatment is live births, it is recommended that in subsequent studies, pregnancy outcomes such as live births, stillbirth, abortion, and multiple pregnancies should be carefully investigated for infertile couples who undergo IUI.

The strength of this study is that it included all sperm parameters before and after processing and the whole process of sperm preparation was performed and reported in the same method by the same team. 

The results of the present study indicate that there was a significant difference in sperm morphology and sperm motility before and after sperm processing between the pregnant and non-pregnant groups. Therefore, it seems that sperm motility and normal sperm morphology have a positive effect on IUI success.

## Figures and Tables

**Table 1:**
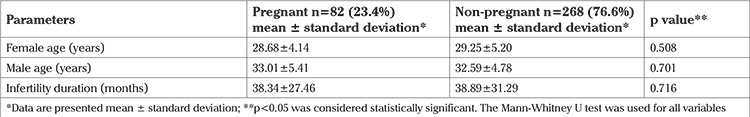
The participants’ demographic data

**Table 2:**
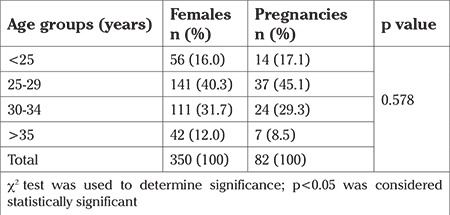
Outcome of intrauterine insemination procedure for different female age groups

**Table 3:**
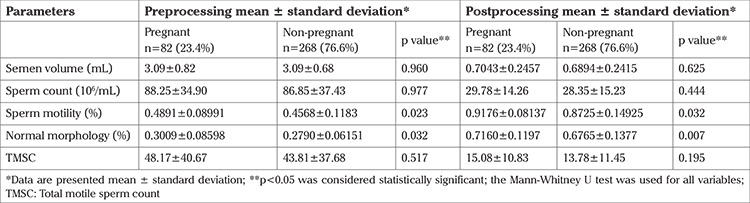
Sperm parameters in the pregnant and non-pregnant groups before and after semen processing

**Table 4:**
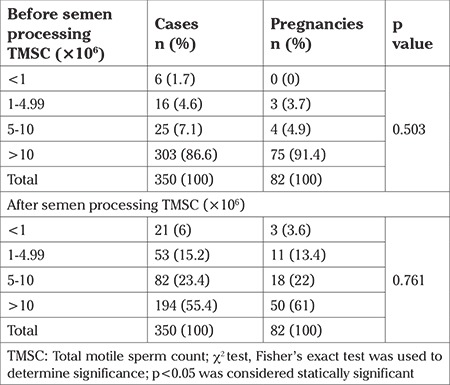
Comparison of pregnancy rates with total motile sperm count before and after sperm processing
